# A hybrid protocol CLAG-M, a possible player for the first-line therapy of patients with mixed phenotype acute leukemia. A Polish Adult Leukemia Group experience

**DOI:** 10.3389/fonc.2024.1395992

**Published:** 2024-05-21

**Authors:** Magdalena Karasek, Anna Armatys, Marek Skarupski, Łukasz Bołkun, Katarzyna Budziszewska, Joanna Drozd-Sokołowska, Ewa Zarzycka, Patrycja Mensah-Glanowska, Małgorzata Gajewska, Janusz Hałka, Agnieszka Kopacz, Witold Prejzer, Olga Chyrko, Tomasz Wróbel, Agnieszka Wierzbowska, Marta Sobas

**Affiliations:** ^1^ Department of Hematology, Blood Neoplasms and Bone Marrow Transplantation, Wroclaw Medical University, Wroclaw, Poland; ^2^ Department of Hematology and Bone Marrow Transplantation, University of Silesia, Katowice, Poland; ^3^ Department of Applied Mathematics, Faculty of Pure and Applied Mathematics, Wroclaw University of Science and Technology, Wroclaw, Poland; ^4^ Department of Mathematics and Computer Science, Eindhoven University of Technology, Eindhoven, Netherlands; ^5^ Department of Hematology, Internal Diseases and Angiology with a Subdivision of Blood Cell Transplantation, University Teaching Hospital in Białystok, Białystok, Poland; ^6^ Department of Hematology, Institute of Hematology and Transfusion Medicine, Warsaw, Poland; ^7^ Warsaw Medical University, Department of Hematology, Oncology and Internal Medicine, Warsaw, Poland; ^8^ Department of Hematology and Transplantology, Medical University of Gdansk, Gdańsk, Poland; ^9^ Department of Hematology, Jagiellonian University, Collegium Medicum in Kraków, Kraków, Poland; ^10^ Department of Internal Medicine and Hematology, Military Institute of Medicine, Warsaw, Poland; ^11^ Department of Hematology and Bone Marrow Transplantology, Clinical Hospital of the Ministry of Internal Affairs and Administration with the Warmia-Mazury Oncology Centre in Olsztyn, Olsztyn, Poland; ^12^ Department of Oncology, University of Warmia and Mazury, Olsztyn, Poland; ^13^ Department of Hematology, University Teaching Hospital them. Fryderyk Chopin in Rzeszów, Rzeszów, Poland; ^14^ Department of Hematology, Medical University of Łódź, Łódź, Poland; ^15^ Department of Hematology, Provincial Multi-specialized Oncology and Trauma Center, Łódź, Poland

**Keywords:** mixed phenotype acute leukemia, ambiguous leukemia, induction treatment, MPAL, hybrid regimen, methylome targeted therapy

## Abstract

**Introduction:**

Mixed-phenotype acute leukemia (MPAL) is a rare disease with poor prognosis. So far, no standard approach has been established as the “know-how” of MPAL is based only on retrospective analyses performed on small groups of patients.

**Materials and methods:**

In this study, a retrospective analysis of the outcomes of adult MPAL patients included in the PALG registry between 2005 and 2024 who received the CLAG-M hybrid protocol as induction or salvage therapy was performed.

**Results:**

Sixteen of 98 MPAL patients received CLAG-M: eight as first-line and eight as salvage therapy. In the first line, two patients achieved partial response (PR), and six achieved complete remission (CR), of whom four successfully underwent allogeneic hematopoietic stem cell transplantation (alloHSCT). Two patients who did not undergo alloHSCT promptly relapsed. Within the whole group, the overall response rate (ORR) was 75% (n = 12/16). With the median follow-up of 13 months, six out of eight patients remain in CR, however, two of them died due to acute graft versus host disease. Out of eight patients who received CLAG-M in the second line, four patients (50%) obtained CR. AlloHSCT was conducted in seven cases, six of which were in CR. Only two patients remained in CR at the time of the last follow-up. Tolerance to treatment was good. The median times for severe neutropenia and thrombocytopenia were 22 days (range, 16–24) and 17 days (range, 12–24), respectively. Overall, grade 3-4 infections were observed in 12 cases, and all infections presented successful outcomes.

**Conclusions:**

CLAG-M is an effective first-line salvage regimen for MPAL with an acceptable safety profile. Early achievement of CR with prompt alloHSCT allows for satisfactory disease control.

## Introduction

Mixed phenotype acute leukemia (MPAL) is a rare disease, representing 2%–5% of acute leukemias (1–3). Contrary to the established lineage-specific antigen expression in acute lymphoblastic leukemia (ALL) and acute myeloid leukemia (AML), MPAL is characterized by blast cells co-expressing lymphoid and myeloid lineage antigens. Due to the heterogeneity of this rare disease and the fact that patients with MPAL are usually excluded from acute leukemia clinical trials, no unified treatment protocol has been established. According to retrospective studies and case reports, the outcomes of therapy based on an ALL-like regimen seem to prevail over those of therapy with an AML-like or hybrid regimen combining both approaches (1, 2, 4–8). Furthermore, the united front is maintained for allogeneic stem cell transplantation (alloHSCT), as outcomes in patients who underwent the procedure are superior to those who received only chemotherapy (3, 4, 9–11).

However, in light of reports implying the possibility of lineage switch (12) and favorable response if treatment matching the DNA methylation patterns of blast cells (13) is applied, the discussion about the most appropriate induction regimen remains doubtful. Uncertainty about introducing therapy based on immunophenotype, cytogenetic, or molecular biology has led to the consideration of a hybrid protocol combining both AML-like and ALL-like regimens. Notwithstanding the studies strongly contradicting that approach (2, 7), some other reports present the benefits of the hybrid regimen (3, 14–16). In accordance with these findings, we retrospectively analyzed MPAL patients who received the hybrid protocol based on cladribine, cytarabine, granulocyte colony-stimulating factor (G-CSF), and mitoxantrone (CLAG-M). Based on the significant research of the Polish Adult Leukemia Group (PALG), which revealed that CLAG-M is a well-tolerated and effective salvage regimen in refractory or relapsed AML (17), we assessed the advantages and safety profile of this treatment protocol in poor-risk MPAL. Furthermore, the prominent effect of cladribine included in treatment protocols for AML, especially in patients with unfavorable cytogenetics and FLT3-ITD mutations, has been proven in previous PALG studies (18–20), which enhanced the advantage of CLAG-M.

Finally, we present a comprehensive analysis of the treatment course and its outcome in MPAL patients treated with the CLAG-M protocol as the first line of induction and salvage therapy.

## Materials and methods

The present study is the outcome of the close cooperation of hematological centers associated with the Polish Adult Leukemia Group, which reported MPAL patients diagnosed and treated with regimens according to the centers’ individual experiences between 2005 and 2024. Of all reported cases, we selected and retrospectively analyzed the group of patients treated with the CLAG-M regimen in the first-line (Group A) of induction and as the salvage protocol (Group B) in case of refractoriness or relapse after the first-line of therapy.

The comprehensive database included information about the patients’ state characterized by age, sex, Eastern Cooperative Oncology Group (ECOG) Performance Status Scale, and presence of hepatosplenomegaly, lymphadenopathy, or central nervous system (CNS) infiltration by leukemia cells. In addition, precise data regarding diagnosis and treatment were collected. The diagnosis was based on blast immunophenotyping according to The European Group for the Immunological Characterization of Leukemias (EGIL) (21)and criteria included in the World Health Organization (WHO) classification and verified according to the WHO 2022 classification (22).

All patients were administered the standard CLAG-M regimen in both induction and salvage protocols, including cladribine at a dose of 5 mg/m^2^ intravenously (iv.) on days 1–5; cytarabine (Ara-C) at a dose of 2,000 mg/m^2^ iv.; on days 1, 2, 3, and 5; granulocyte-colony stimulating factor (G-CSF) 30 MU subcutaneously (sc.) on days 0, 1, 2, 3, 4, and 5, and mitoxantrone at a dose of 10 mg/m^2^ on days 1, 2, and 3 (17). Furthermore, consolidation treatment with a high dose of cytarabine (3,000 mg/m^2^ iv. Every 12 h on days 1, 3, and 5) and intrathecal prophylaxis or treatment with methotrexate (15 mg), cytarabine (40 mg), and dexamethasone(4 mg) were considered. For consolidation treatment, Ara-C was administered at a dosage adjusted for patient age. Patients under 60 years old received high-dose Ara-C (2 g/m^2^–3 g/m^2^ every 12 h i.v. on days 1, 3, and 5), whereas patients above 60 years old received intermediate-dose Ara-C (1 g/m^2^–1.5 g/m^2^ every 12 h i.v. on days 1, 3, and 5). Eventually, all eligible patients were scheduled to undergo alloHSCT. Furthermore, according to the results of cytogenetic analysis, imatinib was included in the treatment of two cases with BCR::ABL rearrangement.

Regarding the high risk of infectious complications associated with intensive treatment, prophylaxis was implemented on the last day of the protocol. The patients were administered levofloxacin (500 mg orally every 24 h), acyclovir (800 orally mg every 12 h), and posaconazole (200 mg orally every 8 h).

Treatment response was assessed according to the ELN criteria (23). To evaluate minimal residual disease (MRD) multiparameter flow cytometry was performed, and quantitative RT-PCR was employed for the t (9, 22) MPAL type.

Furthermore, we analyzed the safety profile of the CLAG-M regimen in first-line of treatment by examining the time to neutrophil and platelet recovery and infectious and non-infectious complications at the time of therapy. Considering the reported prolonged hematological recovery following CLAG-M administration and the consequent heightened risk of infectious complications, two distinct time frames were evaluated for hematological recovery (24). First, the durations of severe neutropenia and thrombocytopenia were evaluated. It was defined as the time from the date of induction implementation to the date of the first stable absolute neutrophil count (ANC) above 500/µL and platelet count above 50,000/µL. Second, the criteria for complete hematological recovery time to ANC >1,000 µL and platelet count >1,000,000 µL were calculated.

Overall survival (OS) was assessed from the date of diagnosis in group A and from CLAG-M administration in group B to the patient’s death or the last follow-up. Eventually, we incorporated the overall response ratio (ORR), defined as the percentage of patients who responded to a CLAG-M regimen with a partial or better response.

### Event rate analysis

First, to evaluate survival, we incorporated Kaplan–Meier analysis. However, due to the small group size and short follow-up time, a specific parametric Weibull model was used to determine whether the risk ratio for each endpoint was constant over time. In this model, the survival function is given as $S\left(t\right)=e^{-{\left(t/\eta \right)}^{\beta }},\;t\geq 0;\;\eta ,\;\beta >0$ ; therefore, the mortality rate is thepower function $h\left(t\right)=\eta^{-\beta }\beta t^{\beta -1}$ . These parameters were estimated using the Maximum Likelihood Method (MLE). The Weibull plot was used to determine whether the dataset followed the Weibull distribution. If 0<β<1, we are in the infant mortality phase; i.e., the mortality rate is decreasing. For β=1 , we deal with random causes of mortality (constant rate), while for β>1 , we deal with aging and increasing rates. The Weibull shape parameter was estimated using 90% confidence intervals (CIs). Due to the small sample size in both groups, we developed a model based on Bayesian methods combined with profile likelihood to increase the statistical power (see **Appendix**). Using Monte Carlo Markov Chain methods, the estimator values and 90% credibility intervals (CrIs) of the shape parameter were determined. To check the similarity between two posterior distributions of beta, we calculated the overlapping index (OI) (25). All calculations were made using R version 4.2.1 in RStudio 2023.06.0. To estimate the parameters, we used the packages WeibullR, surv, survival, flexsurv, and overlapping.

## Results

### The characteristics of patients

Within the presented period, 16 of 98 MPAL patients were treated with the CLAG-M protocol. In eight cases, the CLAG-M regimen was used as the first induction (group A) and also in eight cases as salvage treatment (group B). In group B, CLAG-M was administered after ALL-like or AML-like induction therapy, in three and five cases, respectively. In all cases, there was no response to first-line treatment and all patients were diagnosed with refractory disease.

Half of the patients (n = 8/16, 50%) were diagnosed with B/myeloid type MPAL, six patients in group A and two patients in group B. T/myeloid type MPAL was more frequent in group B, as diagnosed in four patients. One case of MPAL t (9, 22) was reported in groups A and B. Additionally, in group B, B/T/myeloid type MPAL was reported in one case. The median age at diagnosis was 44 years (range, 21–64 years). Nevertheless, the patients in group B were younger, with a median age of 34. The entire research group was characterized by equal numbers of women and men and good performance status in all patients, with a median ECOG score of 1 (range, 0–2). Lymphadenopathy occurred in 43,75% (n = 7/16) of patients and was the most common extramedullary involvement. At the time of diagnosis, each patient underwent lumbar puncture and optional magnetic resonance imaging (MRI) for signs of a cerebral mass. Eventually, one patient was diagnosed with blast cells in the cerebrospinal fluid (CSF) as the only case of central nervous system involvement. Intrathecal chemotherapy was administered twice a week until no blast cells were detected in the CSF, and then twice per consolidation cycle. As for the diagnosis of recurrence, each patient also underwent lumbar puncture and optional MRI, but no CNS involvement was diagnosed.

Half of the patients (n = 8/16, 50%) presented with cytogenetic aberrations; however, karyotype data were missing in three cases in Group B. Complex karyotypes were detected in four cases (n = 4/16, 25%), whereas the other four had single chromosomal aberrations. One case of *BCR-ABL* rearrangement was detected in each group. As far as gene mutations are considered, *RUNX1* and *FLT3-ITD* mutations were most frequent. However, *FLT3-ITD* mutations were only found in Group B. A detailed characterization of the patients is presented in **Table 1**.

**Table 1:** The characteristics of patients.

Number	Sex	Age	MPAL type (WHO 2022)	Blasts in BM smear (%)	Karyotype	Genetic mutations
Patients who received CLAG-M in the first line of induction						
1.	Female	51	MPAL t(9;22)	92.5	46 XX, t(9;22) [85%]	None
2.	Male	64	B/Myeloid	83	46, XY, t(8;21) del(9)/ 45, XY-, t(8;21), del(9)	*RUNX1*
3.	Male	37	B/Myeloid	73	46, XY	None
4.	Male	56	B/Myeloid	56	47, XY +8, t(8;21), del 11	*RUNX1*
5.	Male	59	T/Myeloid	48	42~48, XY,del(9)(p21)[5],+13[4],+mar1[6],+mar1x2[2],+mar2[8],+mar3[5][cp13]/46,XY[7]	*CEBPA*
6.	Female	43	B/Myeloid	50.7	46, XY	None
7.	Female	35	B/Myeloid	78	46,XX,del(1)(p22p11),del(2)(q33)[10],add(3)(q23)[10],der(9)del(9)(p22)add(9)(q22),add(16)(p13.3)[5],-17,der(18)t(17;18)(q11.1;q12.2),+22[4],+mar[5][20]	None
8.	Female	63	B/Myeloid	76	46, XX, t (3;8)/46, XX	None
Patients who received CLAG-M as salvage treatment						
1.	Female	28	B/T/Myeloid	81	46, XY, del13(q12.31-33)	*RUNX1*
2.	Female	33	T/Myeloid	90	No data	No data
3.	Male	63	T/Myeloid	86.5	No data	No data
4.	Male	21	B/Myeloid	68	46, XX	None
5.	Female	36	T/Myeloid	76	No data	*FLT3-ITD*
6.	Male	45	MPAL t(9;22)	89	No metaphases	None
7.	Female	31	T/Myeloid	90	47, XY+5, t(6;14)(p21,q32)[9]	*FLT3-ITD*
8.	Male	63	B/Myeloid	56.4	46, XX [20]	*FLT3-ITD*

BM, bone marrow.

### The safety of treatment

The patients presented with severe neutropenia for a median of 22 days (range, 16–24 days) in Group A and 24 days (range, 18–27 days) in Group B, and severe thrombocytopenia for a median of 17 days (range, 12–24 days) in Group A and 20 days (range, 18–25 days) in Group. B. Regarding complete hematological recovery, half of the patients in Group A met the criteria. Of the eight patients in this group, seven (87.5%) had platelet counts > 100,000/µL, with a median thrombocytopenia duration of 25 days (range, 19–44). Additionally, four patients (50%) had ANC levels >1,000/µL, with a median time to neutrophil recovery of 25.5 days (range, 23–28 days).

Regarding infectious complications, the incidence and classification according to the Common Terminology Criteria for Adverse Events (CTCAE version 5.0) were similar in both groups: six (75%) cases of grade 3–4 infection in each group. In all cases, the epidemiological factor was the bacterium, and all had successful outcomes. All infections were reported within an ANC >500/µL. No noninfectious complications were observed. Finally, neither admission to the intensive care unit nor death during induction treatment was observed in either group.

### The results of treatment with CLAG-M

Six of eight patients (75%) treated with CLAG-M as the first line of induction achieved complete remission (CR); however, three of them had partial hematologic recovery (CR_h_). The remaining two patients were evaluated for partial response (PR). Additionally, 67% (n = 4/6) of CR patients had negative MRD. The outcome of CLAG-M as salvage treatment was less prominent, as only 50% (n = 4/8) of the patients achieved CR. Unfortunately, data on MRD in Group B are lacking. Finally, regarding CLAG-M administration, the ORR were 75% (n = 12/16), 100% in Group A and 50% in Group B (n = 4/8).

Regarding further treatment, in Group A, patients in CR and CR_h_ were prioritized for alloHSCT after a consolidation cycle with a high or intermediate dose of cytarabine, depending on whether the patient was under or over 60 years old, respectively. Two patients in PR received the therapy based on the best physician experience; in one case, another cycle of CLAG-M and hyperCVAD in another case. At the observation endpoint, both were evaluated for CR2 with complete hematological recovery, MRD negative and positive, respectively.

In Group A all patients were considered eligible for alloHSCT, and successful qualification was performed in seven cases (87.5%) (five in CR1 and two in CR2), as one patient did not consent to the procedure. When the study endpoint was reached, the procedure was performed in four patients who remained in CR1 before allotransplantation. In all cases, the conditioning regimen before alloHSCT was preferably based on a combination of chemotherapy and radiotherapy. One patient in CR1 eventually did not undergo alloHSCT due to the lack of a matching donor. Two patients in CR2 awaited allo-HSCT, as the matching donor was confirmed. Two patients, who did not receive allografts promptly after consolidation, relapsed. They qualified for salvage treatment with azacytidine and venetoclax, with no response. Eventually, both patients died due to relapse and refractory disease. Two more deaths were reported in Group A, both due to complications of acute graft versus host disease (aGvHD).

In Group B, allo-HSCT was performed in seven cases (87.5%), including five with CR and two with refractory disease. The conditioning regimen prior to alloHSCT was similar to that Group A and was based on a combination of chemotherapy and radiotherapy. In Group B, six patients (75%) died at the time of follow-up. In four of them, the cause was the underlying disease: primary resistance and relapse after alloHSCT, each in two cases. The other two patients died of infections.

After a median follow-up time of 13 months (3–131), 10 of 16 (63%) patients died, six due to relapse or refractory disease, four from infection or complications after alloHSCT. In Group A, regardless of deaths due to GvHD complications, six out of eight (75%) patients remained in CR. The median OS was 9 months (range, 3–131 months). In Group B, two patients remained in CR after alloHSCT, with an OS of 11 and 23 months. The median OS was 21.5 months (range, 3–28 months). Details about the treatment outcomes in Groups A and B are presented in **Tables 2**, **3**.

**Table 2:** The treatment outcome in patients treated with CLAG-M in the first line of induction.

Patient number	Induction	Treatment response	MRD status	Consolidation	CNS prophylaxis with i.th.ch.	AlloHSCT	Relapse	Relapse or refractoriness therapy	Death	Response state at the time of FU	OS (months)
1.	CLAG-M + imatinib	CR	Negative	HD-AraC	Yes	Yes	No	No	No	CR	131
2.	CLAG-M	CR_h_	Positive	ID-AraC	Yes	Yes	No	No	No	CR	15
3.	CLAG-M	CR_h_	Negative	HD-AraC	Yes	Yes	No	No	Yes, due to bleeding in course of GvHD	CR	8
4.	CLAG-M	CR_h_	Negative	HD-AraC	Yes	Yes	No	No	Yes, due to sepsis in course of GvHD	CR	9
5.	CLAG-M	CR	Negative	HD-AraC	No	Not done due to the lack of patient’s consent	Yes	AZA+VEN	Yes, due to relapse	Relapsed disease	10
6.	CLAG-M	CR	Positive	HD-AraC	No	Not done due to donor-matching failure	Yes	AZA+VEN	Yes, due to relapse	Relapsed disease	6
7.	CLAG-M	PR (from 78% to 6% of blasts)	Not applicable	Not applicable	Yes	Will be performed after reinduction with CLAG-M	No	CLAG-M	No	CR, MRD (-)	3
8.	CLAG-M	PR (from 76% to 23% of blasts)	Not applicable	Not applicable	Yes	Will be performed after reinduction with hyperCVAD	No	HyperCVAD	No	CR, MRD (+)	5

CLAG-M, cladribine, cytarabine, granulocyte colony-stimulating factor; CR, complete response; MRD, minimal residual disease; CNS, central nervous system; i.th.ch, intrathecal chemotherapy; alloHSCT, allogeneic hematopoietic stem cell transplantation; OS, overall survival; CR, complete response; HD-AraC, high dose cytarabine; AZA + VEN, azacytidine + venetoclax; GvHD, graft versus host disease.

**Table 3:** The treatment outcome of patients treated with CLAG-M in refractory/relapsed MPAL.

Patient number	1st line regimen	Response to 1st line treatment	2nd line regimen	Response to 2nd line treatment	3rd line regimen	Response to 3rd line treatment	alloHSCT	Relapse after alloHSCT	Death	Response state at the time of FU	OS (months)
1.	PALG ALL7 Ph (−)<55 y.o.	Refractoriness	CLAG-M	CR	No	–	Yes	Yes	Yes, due to infection	CR after second alloHSC	28
2.	DAC	Refractoriness	CLAG-M	CR	No	–	Yes	Yes	Yes, due to relapse	Relapsed disease	23
3.	mini FLAM	Refractoriness	CLAG-M	CR	No	–	Yes	Yes	Yes, due to infection	PR after FLAG-IDA therapy	23
4.	DA (2 + 5)	Refractoriness	CLAG-M	Refractoriness	No	–	No	Not applied	Yes, due to refractory disease	Refractory disease	2
5.	DA + midostaurin	Refractoriness	CLAG-M	Refractoriness	HyperCVAD	CR	Yes	No	No	CR	11
6.	PALG ALL7 Ph (-)< 55 y.o. + imatinib	Relapse	CLAG-M + dasatinib	CR	No	–	Yes	No	No	CR	23
7.	DA	Refractoriness	CLAG-M	Refractoriness	No	–	Yes in active disease	Progression	Yes, due to refractory disease	Refractory disease	9
8.	DA + midostaurin	Refractoriness	CLAG-M	Refractoriness	VEN+AZA	CR	Yes	Yes	Yes, due to relapse	Relapsed disease	20

PALG ALL7 Ph (−)<55 y.o. – treatment protocol by Polish Adult Leukemia Group in acute lymphoblastic leukemia chromosome Philadelphia negative: dexamethasone, vincristine, daunorubicin, pegaspargase; DAC, treatment protocol by Polish Adult Leukemia Group: daunorubicin, cytarabine, cladribine; miniFLAM, fludarabine, cytarabine, mitoxantrone (reduced intensity); DA, daunorubicin, cytarabine; CLAG-M, cladribine, cytarabine, granulocyte colony-stimulating factor, mitoxantrone; hyperCVAD, cyclophosphamide, vincristine, doxorubicin, dexamethasone; VEN + AZA, venetoclax, azacytidine; alloHSCT, allogeneic hematopoietic stem cell transplantation; CR, complete response; OS, overall survival.

### Survival analysis

**Figure 1:**
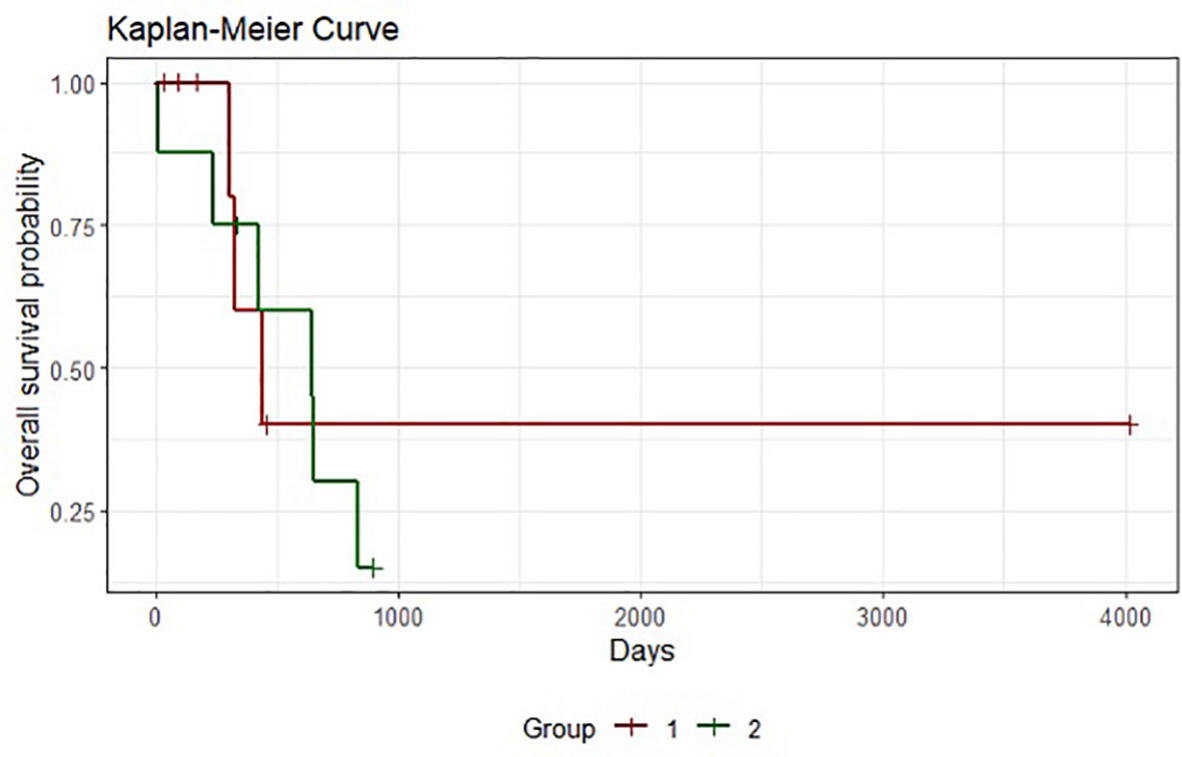
Comparison of survival in Groups (A, B). Kaplan–Meier plot.

**Figure 2:**
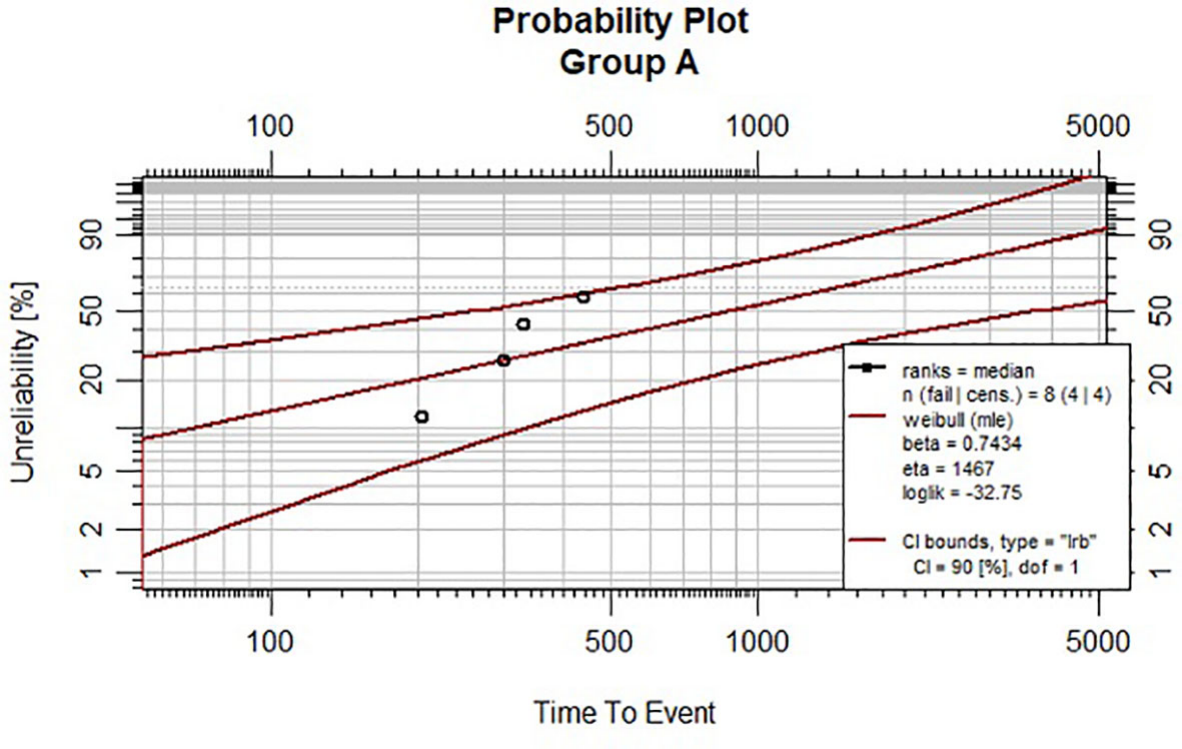
Weibull plot with 90% CIs for Group (A).

**Figure 3:**
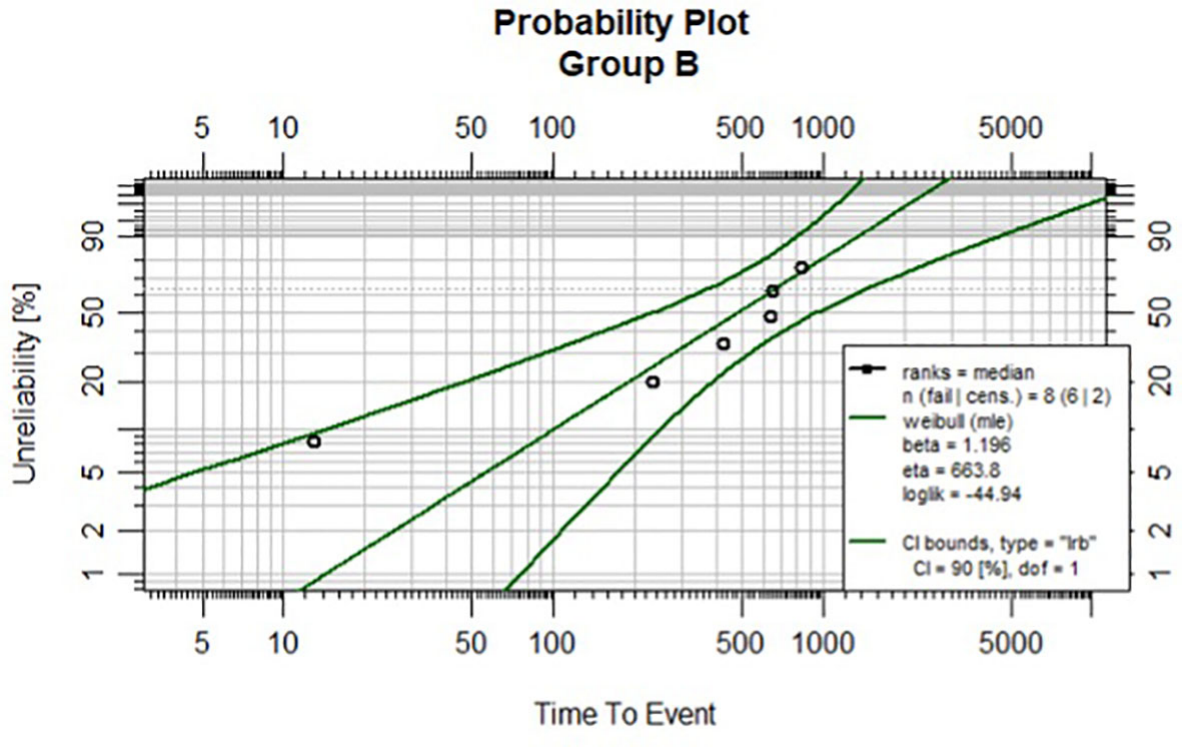
Weibull plot with 90% CIs for Group (B).

Survival assessment using the Kaplan–Meier plot is presented in **Figure 1**. Regarding the outcomes of the individually created statistical model, we compared the shape factor, β. Probability plots with 90% Cis are presented in **Figures 2**, **3** for Groups A and B. Note that all the points are within the confidence bounds. The numerical results are listed in **Table 4**.

**Table 4:** The numerical results of Weibull model for group A and B.

Estimates:	Group A					
	Est		L90%	U90%	se	
shape	0.743		0.416	1.326	0.262
scale	1467.49		482.02	4472.71	994.33
N = 8	Events: 4			Censored: 4		
Total time at risk: 5877 (days)				AIC = 69.49478	
Log-likelihood = -32.7474				df = 2	
Estimates:	Group B					
	Est	L90%		U90%	se	
shape	1.196	0.653		2.194	0.629	
scale	663.84	378.69		1164.32	226.70	
N = 8	Events: 6			Censored: 2		
Total time at risk: 4028 (days)				AIC = 93.88921		
Log-likelihood = -44.9446				df = 2		

In both groups, the null hypothesis cannot be rejected. The following results were obtained.

In Group A: β = 0.7078, CrI = (0.3182; 1.1756), while in Group B: β = 1.2103, CI = (0.5703; 1.9913).

In Group B, β indicates that we deal with an increasing death rate (since β >1). However, in Group A estimation of β indicates that the death rate is decreasing (since β<1). The overlapping index (OI) is also calculated. Overlapping can be used to assess the posterior distribution of a Bayesian model. In our case OI = 0.55. This results in a 45% difference between the posterior distributions of the shape parameters.

## Discussion

The presented overview analyses of a homogenous group of adults, whose age is predominantly over their 40s. Regardless of age, sex, and MPAL type (B/mielo, T/mielo, or others), 75% of CR was accomplished with CLAG-M in the first-line of induction accompanied by low non-hematological toxicity and no prolongation of severe neutropenia or severe thrombocytopenia. As far as meta-analysis and most treatment guidelines preferably recommend ALL regimens for MPAL treatment (1, 2, 4–8), selective treatment may lead to clonal expansion of blasts resistant to initial lineage-based chemotherapy. The substantiation of these doubts could be found in the recommendations for administering an AML-based regimen if no response to ALL-based treatment was observed (4, 26). Furthermore, MPAL-like phenomena of lineage switches cannot be neglected. The hypothesis presented by Hu et al. implies that leukemic clones involved in lineage switching may be derived from multipotent hematopoietic cells (27). Moreover, the pressure of ALL-like treatment has been reported to be a potential cause of lineage switching. According to published data, CD19 targeted therapy of B-ALL with blinatumomab or αCD19 CAR-T cells may lead to myeloid switch (28–30), and B-cell precursor ALL is prone to myeloid switch under standard intensive ALL-like treatment (31, 32). These phenomena were additionally associated with alternations in transcription factors, such as Pu1 and Pax5, which have also been described as potential causes of lineage switch (33). Considering these findings, hybrid protocols may be a solution to prevent lineage switching.

Nevertheless, previously reported hybrid protocols combining regimens from both ALL and AML protocols are too toxic (2, 7). Therefore, we opted for a CLAG-M regimen characterized by less toxicity and a more multidirectional profile of effectiveness that may overcome the challenges arising from the complex pathogenesis of MPAL without the potential selection of any subclones. To date, the CLAG-M protocol has been widely reported to be beneficial in AML with poor prognosis, but has also proven to be well-tolerated (17, 34–36).

In terms of multidirectional activity, agents in the CLAG-M regimen have significant cytotoxic effects on both myeloid and lymphoid lineages. Their administration is an effective approach in high-risk AML (37) and relapsed or refractory ALL (38), whereas cladribine is commonly administered for the treatment of lymphoma. Another compound in the CLAG-M regimen, mitoxantrone, may be used in both AML and ALL treatment protocols. Mitoxantrone is a DNA-damaging agent whose association with Ara-C and cladribine results in the synergistic inhibition of DNA repair mechanisms (17, 39). The addition of G-CSF potentiates Ara-C sensitivity, especially in cells with low proliferative activity, thereby enhancing treatment response (40). Cladribine increases the cellular uptake of Ara-C and potentiates its intracellular metabolism, thereby intensifying the cytostatic effect (41–43). Furthermore, cladribine actively inhibits DNA synthesis by incorporating it into DNA strands and directly damaging the mitochondrial membrane, leading to cell apoptosis (44).

Cladribine has also been reported to have hypomethylating activity (45), which was proven in previous studies by Libura et al. (46). Patients diagnosed with AML and coexisting *IDH1/2* mutations leading to DNA hypermethylation and epigenetic dysregulation had more successful outcomes when cladribine was applied in the induction protocol. Based on the favorable results of the aforementioned study, cladribine may be characterized as a crucial agent of the regimen, especially since the investigation by Alexander et al. (47) reported a significant contribution of the methylation profile in MPAL pathogenesis. Furthermore, according to Takahashi et al., achieving a complete response in MPAL is more likely if AML-like or ALL-like regimens are administrated according to the methylation profile presented by blasts (13). Since methylome examination is not a standard diagnostic procedure, it could be challenging to use it as an eligibility criterion for treatment. Thus, applying the general hypomethylating agent, cladribine, may restrict methylation changes in lineage-defining transcription factor genes responsible for mixed immunophenotype presentation (13). Eventually, its general effects may limit lineage-specific clone selection and resistance to therapy.

Nevertheless, decisions regarding further treatment in patients who achieve PR remain a challenging clinical problem. So far, the literature and physicians’ experiences suggest that the next line of treatment should be switched to a regimen specified for another lineage than the previous one (48–50). Such a procedure can be justified if lineage-specific treatment is administered; however, in the first-line treatment, we recommend a hybrid regimen with a broader cytotoxic effect involving both lineages. Thus, in light of insufficient literature data on the management of PR patients who have received hybrid protocols, we advocate CLAG-M as a reinduction.

Notwithstanding the broad cytostatic effect of CLAG-M on leukemia cells, the impact of some mutations on the course of the disease, and thus treatment, cannot be neglected. Interestingly, cladribine was reported to overcome the negative effect of *FLT3-ITD* mutation and improve treatment response in patients diagnosed with AML *FLT3-ITD* positive (18). A similar effect is believed to be observed in patients with MPAL *FLT3-ITD* positivity, which implies an additional advantage of the CLAG-M protocol. Nevertheless, the administration of tyrosine kinase inhibitors (TKI) such as imatinib and dasatinib in cases of MPAL with BCR-ABL rearrangement, has a significant impact on improving the prognosis of patients’ lifespans (4, 51).

In this analysis, we proved that no prolongation of severe cytopenia was observed in either research group. Although partial hematological recovery was reported in half of the CR patients in Group A, all infectious complications occurred during the period of severe cytopenia and were successfully treated with empirical and targeted antibiotic therapy; thus, no admission to the intensive care unit was needed. All patients were able to continue further treatment safely without significant interruptions due to significant hematological toxicity of the protocol or prolonged serious infections. The tolerance of CLAG-M in the second-line treatment in patients who already received intensive regimens was similar to that in the first-line treatment. However, infectious prophylaxis and strict clinical supervision are necessary because most patients suffer from infectious complications. Targeted infectious therapy is likely to result in a successful outcome, even in elderly patients, as proven in our study.

Discussions about MPAL treatment, including alloHSCT, must be addressed. The outcomes of retrospective studies have demonstrated the beneficial role of alloHSCT. In a report by Heesch et al., the 5-year survival rate of patients with MPAL who underwent the transplant procedure was 70% compared to 19% for those who received only chemotherapy (11). Favorable results of alloHSCT in MPAL were also demonstrated by Munker et al. (3-years OS 56.3%) (9), Shimizu et al. (5-years OS 48%) (51), and Liu et al. (3-years OS 45%) (10). It is worth mentioning that MRD-negative CR achieved by CLAG-M treatment significantly increased the chances of success of the procedure and preserved reasonable disease control. CR obtained with CLAG-M in the first line of induction in most patients allowed four of them to receive allograft quickly and consolidate the treatment response. Nevertheless, deferring alloHSCT for a disease with such a high risk and unfavorable prognosis as MPAL can lead to rapid relapse, as revealed by the case of two patients in Group A. Nowadays, improvements in alloHSCT methodologies and post-transplant care broads patients eligible for allotransplantation, and age and comorbidities no longer present strict limitations (52). Taking this advantage, MPAL patients should be widely qualified for alloHSCT as the greatest change for good disease control and prolonged survival.

On the other hand, regarding alloHSCT outcomes in patients who received CLAG-M as salvage therapy, complete response was maintained in two cases. It is clear that, although statistically irrelevant in clinical practice, patients who undergo alloHSCT in CR1 are more likely to remain in remission. These data contradict the report of Munker et al., who demonstrated no difference in outcomes of alloHSCT between MPAL patients who underwent the procedure in CR1 or CR2. However, these results were not statistically relevant (9). However, further studies are required. Nevertheless, our study is illustrated comparatively to the general analysis of OS after alloHSCT in CR1, CR2, and no response by Bolo et al. (53).

The limitation of this small research group required the development of an individual mathematical model to assess the significance of the impact of CLAG-M on MAPL patient survival. In Group B, the mortality increasing tendency remained unaffected, whereas the death rate was reduced in Group A, which received CLAG-M as the first line, thereby achieving mostly CR. Thus, the differential factor between the two groups can be indicated as an effective intensive regimen with a broad cytotoxic action in induction therapy, which allows for a good response and prompt alloHSCT. Ultimately, the CLAG-M regimen as the first-line treatment may offer a significant opportunity for consolidation treatment and lifespan prolongation. Considering the limitations of this research, we hope for international multicenter cooperation to revise the prepared mathematical model and establish an opportune therapeutic protocol for patients with this rare disease with an adverse prognosis.

## Conclusions

Following our findings, we opted for intensive yet acceptable safety profile induction with the hybrid protocol of CLAG-M, which, with prompt alloHSCT, allows for reasonable disease control. All patients included in the study were able to receive CR in the first line of induction and proceeded to alloHSCT with no deaths or serious complications. The protocol is intensive but still well-tolerated, and in the case of a disease with such complex pathogenesis as MPAL, treatment with a broad profile of cytostatic action such as the CLAG-M regimen can improve the chances of achieving CR. Undoubtedly, our study is limited by the group size, comparability, well-documented nature, and short follow-up time. Nevertheless, this study sheds light on possible approaches to MAPL treatment. Further studies on the mechanisms underlying MPAL transformation and ambiguous phenotypes are crucial for a better understanding of the course of the disease and possible targeted therapy. However, until then, CLAG-M protocols will be a promising treatment scheme for MPAL patients to improve their therapy results.

## Data availability statement

The original contributions presented in the study are included in the article/**Supplementary Material**. Further inquiries can be directed to the corresponding author.

## Ethics statement

The studies involving humans were approved by the local Bioethics Committee of Wroclaw Medical University. The studies were conducted in accordance with the local legislation and institutional requirements. The participants provided their written informed consent to participate in this study.

## Author contributions

MK: Conceptualization, Formal analysis, Investigation, Methodology, Project administration, Validation, Visualization, Writing – original draft, Writing – review & editing. AA: Investigation, Writing – review & editing. MSk: Data curation, Formal analysis, Methodology, Software, Visualization, Writing – original draft, Writing – review & editing. ŁB: Investigation, Writing – review & editing. KB: Investigation, Writing – review & editing. JD-S: Investigation, Validation, Writing – review & editing. EZ: Investigation, Writing – review & editing. PM-G: Investigation, Writing – review & editing. MG: Investigation, Writing – review & editing. JH: Investigation, Writing – review & editing. AK: Investigation, Writing – review & editing. WP: Investigation, Writing – review & editing. OC: Investigation, Writing – review & editing. TW: Supervision, Validation, Writing – review & editing. AW: Methodology, Supervision, Validation, Writing – review & editing. MSo: Methodology, Project administration, Supervision, Validation, Writing – review & editing.
