# Supplementary material for: A hybrid protocol CLAG-M, a possible player for the first-line therapy of patients with mixed phenotype acute leukemia. A Polish Adult Leukemia Group experience
Source: Front Oncol. 2024 May 21;14:1395992. doi: 10.3389/fonc.2024.1395992 (PMC11148324; doi:10.3389/fonc.2024.1395992)
Supplement: Supplementary file 1 [file DataSheet_1.docx]

**Appendix**

Let us assume that we have a random sample of censored failure reporting times $X_{1},\ldots, X_{n}$, where $X_{i}=\min\{T_{i},C\}$ , $T_{i}\sim Weibull \left( \eta, \beta\right)$ are the failure reported time of events with Weibull survival function $S\left( t \right)=e^{-}\left( t/\eta\right)^{\beta}$ and probability density function $f\left( t \right)= -S^{'}\left( t \right); \eta,\beta>0$, $C>0$ is the end time of the observation (censoring time). Let us order the sample so that $X_{(1)}\leq X_{\left( 2 \right)}\leq\ldots\leq X_{(n)}$ and suppose that we observe $m-n$ censored data (i.e. $m$ uncensored). Then the likelihood function for this model is

$$L\left( x_{1},\ldots, x_{n} \right|\eta, \beta)=\prod_{i=1}^{m} f(t_{(i)})\cdot S^{n-m}\left( C \right)$$

Taking logarithm we obtain the loglikelihood function

$$l\left( x_{1},\ldots, x_{n} | \eta,\beta\right)=m\log(\beta)-m\beta\log\left( \eta\right)+\left( \beta-1 \right)\sum_{i=1}^{m} \log\left( t_{\left( i \right)} \right)-\eta^{-\beta}\sum_{i=1}^{n} x_{\left( i \right)}^{\beta}$$

First, we will maximize the above function with respect to the $\eta$ variable. Equating the first partial derivative with respect to eta to 0, we obtain the following form of the estimator

$$\hat{\eta}=\left( \frac{\sum_{i=1}^{n} x_{\left( i \right)}^{\beta}}{m} \right)^{\frac{1}{\beta}}$$

By inserting it into the above equality, we obtain the profile likelihood function

$$L_{p}\left( x_{1},\ldots, x_{n} | \beta\right)\propto\beta^{m}\left( \sum_{i=1}^{n} x_{\left( i \right)}^{\beta} \right)^{-m}\left( \prod_{i=1}^{m} t_{\left( i \right)} \right)^{\beta-1}$$

Now let us apply Bayesian methodology. Let $\pi_{priori}(\beta)$ be a priori distribution for shape parameter. Since we do not have any additional knowledge about the observed events further we will assume non-informative prior, i.e. $\pi_{priori}\left( \beta\right)\propto\frac{1}{\beta}$. Then, using Bayes rule the posterior distribution for shape parameter has form

$$\pi_{postetior}\left( \beta\right)\propto\beta^{m-1}\left( \sum_{i=1}^{n} x_{\left( i \right)}^{\beta} \right)^{-m}\left( \prod_{i=1}^{m} t_{\left( i \right)} \right)^{\beta}$$

The Bayesian estimator  $\hat{\beta}_{SE}$ is the expected value of the posterior distribution. In the same spirit, we construct the $1-p$ confidence intervals by finding $\frac{p}{2}$and $1-\frac{p}{2}$ quantiles from the posterior distribution. Finding the analytical form of the estimator is not possible in this case, hence the Monte Carlo Markov Chain, in particular the Metropolis-Hasting Algorithm, will be used with 100000 iterations (1000 burn-in samples).
